# Monitoring and predicting land use and land cover changes using remote sensing and GIS techniques—A case study of a hilly area, Jiangle, China

**DOI:** 10.1371/journal.pone.0200493

**Published:** 2018-07-13

**Authors:** Chen Liping, Sun Yujun, Sajjad Saeed

**Affiliations:** State Forestry Administration Key Laboratory of Forest Resources & Environmental Management, Beijing Forestry University, Beijing, China; University of Copenhagen, DENMARK

## Abstract

Land use and land cover change research has been applied to landslides, erosion, land planning and global change. Based on the CA-Markov model, this study predicts the spatial patterns of land use in 2025 and 2036 based on the dynamic changes in land use patterns using remote sensing and geographic information system. CA-Markov integrates the advantages of cellular automata and Markov chain analysis to predict future land use trends based on studies of land use changes in the past. Based on Landsat 5 TM images from 1992 and 2003 and Landsat 8 OLI images from 2014, this study obtained a land use classification map for each year. Then, the genetic transition probability from 1992 to 2003 was obtained by IDRISI software. Based on the CA-Markov model, a predicted land use map for 2014 was obtained, and it was validated by the actual land use results of 2014 with a Kappa index of 0.8128. Finally, the land use patterns of 2025 and 2036 in Jiangle County were determined. This study can provide suggestions and a basis for urban development planning in Jiangle County.

## Introduction

Land use research programs at a global scale have become central to international climate and environmental change research since the launch of land use and land cover (LULC) change project[1]. LULC has two separate terminologies that are often used interchangeably[2]. Land cover refers to the biophysical characteristics of earth’s surface, including the distribution of vegetation, water, soil, and other physical features of the land. While land use refers to the way in which land has been used by humans and their habitat, usually with an emphasis on the functional role of land for economic activities[3–5]. For instance, in terms of urbanization, a large amount of agricultural / forestry land has been transformed into urban land, and mining activities / oil exploitation have occurred worldwide to meet the demands of people and can directly and obviously lead to the LUCC[6, 7]. In past studies, global environmental changes such as emissions of greenhouse gases, global climate change, loss of biodiversity, and loss of soil resources have been closely linked to LULC changes[8]. Land use and land cover change (LULCC) is the conversion of different land use types and is the result of complex interactions between humans and the physical environment[9]. LULCC is a major driver of global change and has a significant impact on ecosystem processes, biological cycles and biodiversity[7, 10, 11]. Moreover, LULCC is also closely related to the sustainable development of the social economy[12, 13]. Vast areas of the earth’s terrestrial surface have undergone LULCC[14–16]. With rapid economic development, land uses change more rapidly, and the contrast among land use types also increases[17].

Various techniques of LULC change detection analysis were discussed by Lu *et al*[18]. It is possible to establish a model to predict the trends in land uses in a certain period of time through the study of past land use changes, which could provide some basis for scientific and effective land use planning, management and ecological restoration in a study area and guidance for regional socio-economic development. Therefore, accurate and up-to-date land cover change information is necessary for understanding and assessing LULC changes. Remote sensing (RS) and geographic information system (GIS) are essential tools in obtaining accurate and timely spatial data of land use and land cover, as well as analyzing the changes in a study area[19–21]. Remote sensing images can effectively record land use situations and provide an excellent source of data, from which updated LULC information and changes can be extracted, analyzed and simulated efficiently through certain means[22, 23]. Therefore, remote sensing is widely used in the detection and monitoring of land use at different scales[24–27]. GIS provides a flexible environment for collecting, storing, displaying and analyzing digital data necessary for change detection[19, 28, 29].

Land cover change modeling means time interpolation or extrapolation when the modeling exceeds the known period[30]. Commonly used models for estimating land cover changes are analytical equation-based models[31], statistical models[32], evolutionary models[33], cellular models[34], Markov models[35], hybrid models[36], expert system models[37] and multi-agent models[38]. At present, the most widely used models in land use change monitoring and prediction are cellular and agent-based models or the mixed model based on these two types of models[39–42]. The Markov chain and Cellular Automata (CA-Markov) model, one of a mixed models, is the hybrid of the Cellular Automata and Markov models. This model effectively combines the advantages of the long-term predictions of the Markov model and the ability of the Cellular Automata (CA) model to simulate the spatial variation in a complex system, and this mixed model can effectively simulate land cover change[43]. A CA model is a dynamic model with local interactions that reflect the evolution of a system, where space and time are considered as discrete units, and space is often represented as a regular lattice of two dimensions[44]. CA-based models have a strong ability to represent nonlinear, spatial and stochastic processes. However, CA model cannot represent macro-scale social, economic and cultural driving forces that influence urban expansion well. Thus, an integration of agents into CA models results in the improved CA-Markov model[40]. In the Markov model, the change in an area is summarized by a series of transition probabilities from one state to another over a specified period of time. These probabilities can be subsequently used to predict the land use properties at specific future time points[45]. The use of the CA-Markov model in LULCC studies has advantages such as its dynamic simulation capability; high efficiency with data, scarcity and simple calibration; and ability to simulate multiple land covers and complex patterns[17, 46]. Many researchers have applied the CA-Markov model to monitor land use and landscape changes and predictions[23, 36, 47, 48]. Therefore, we adopted this method to obtain reliable results for Jiangle. In this study, the 2025 and 2036 LULCs were predicted based on the state of 2003 and 2014 LULCs.

In recent decades, rapid population migration from rural to urban regions and improved economic conditions in China have resulted in unprecedented LULC changes and urban expansion rates[6]. Drivers of urbanization and changes in urban planning should be taken into account[48]. Many studies have focused on the LULCC at the scale of large cities to provinces in terms of surface runoff, urban impervious surface, surface urban heat islands, etc.[1, 6, 8, 49, 50]. While there are few studies on small cities such as Jiangle, a county that is west of Fujian Province. Fujian Province, one of the most economically developed provinces in China and located in the southeastern hilly area, plays an important role in China. Moreover, Fujian Province is the core area of the “the Belt and Road Initiative” policy[51]. Jiangle is the representative county of the hilly area and owns a state-owned forest farm. In the “National Wood Strategic Reserve Production Base Construction Plan (2013–2020)”, Jiangle is one of the bases. Therefore, understanding the LULC in this area and predicting future LULC can be of great importance.

This study seeks to utilize remotely sensed data and GIS tools to analyze the LULCC in Jiangle County in Fujian, China for the purpose of detecting changes in the area by comparing images between two years. Based on the Markov model, the transfer probability was established based on the data from 1992 and 2003, and the predicted data of 2014 was processed using the transfer probability and suitability maps in the CA model. After validation, the land use and land cover in 2025 and 2036 were predicted. Finally, a scientific basis for decision-making for the region's ecological protection and optimal allocation of resources is provided.

## Study area

**Fig 1:**
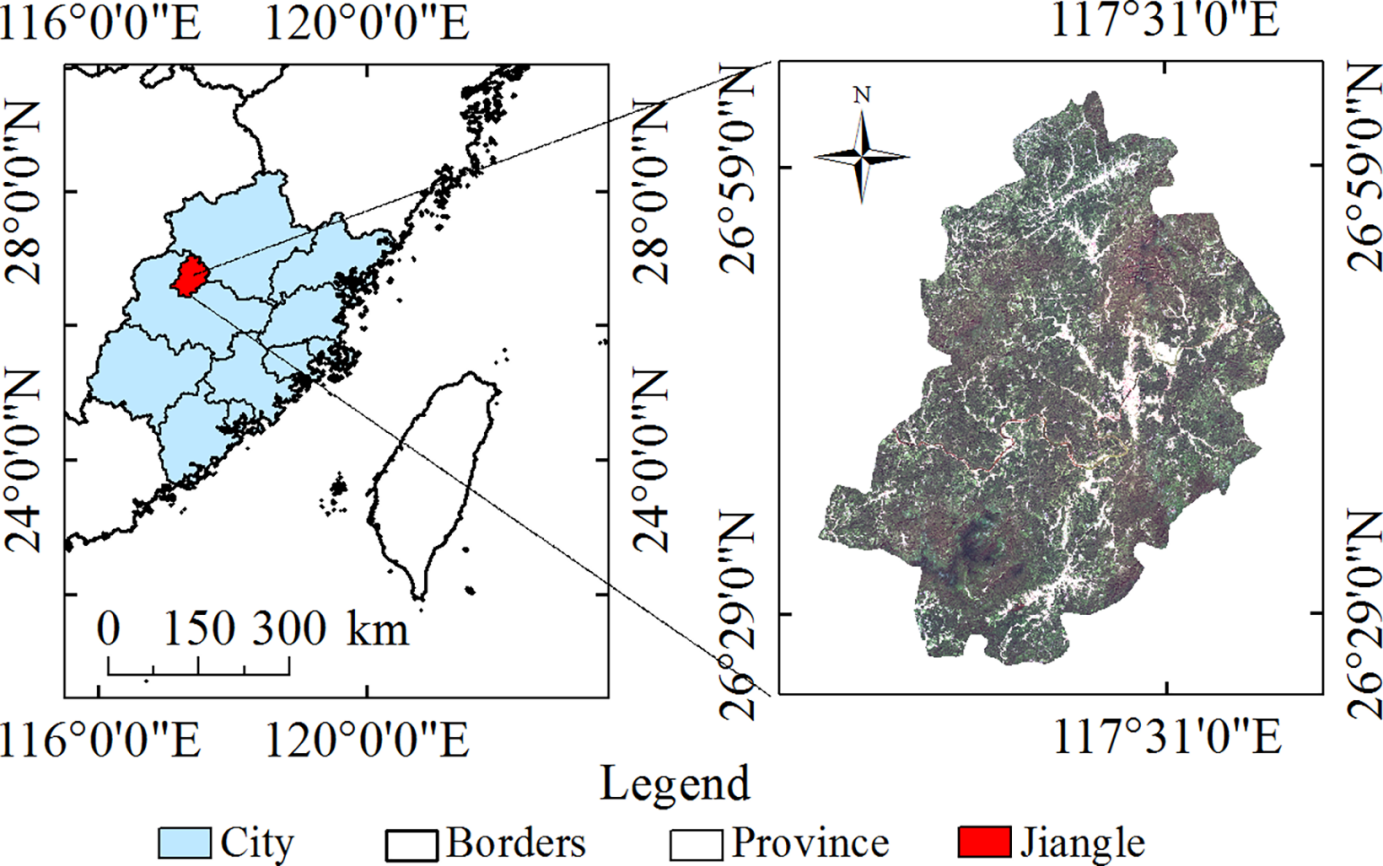
Schematic diagram of the study area (for illustrative purposes only, the image here is from the Landsat8 OLI data of 2014).

Jiangle, located in the western part of Fujian Province, has a latitude between "26°25’ 31”~27°4’8” N and a longitude between 117°5’2”~117°39’56”E (Fig 1). The study area is in the subtropical monsoon climate zone, with marine and continental climate characteristics. The annual average temperature is 18.7°C. The annual average rainfall during 2011 to 2015 was 1802.16 mm, and the frost-free period is approximately 273 days[52]. The precipitation during April—August accounts for more than 60% of the annual precipitation. The study area is in the middle of the main section of the Wuyi Mountains, with an average elevation of 540 m, and its highest peak in the southwest is Longxi Mountain with a height of 1620 m. The altitude in the center of the study area is lower than the altitude around the edge of the area. The terrain is tilted from northwest to southeast. The terrain is complex, and 90% of the region is the mountain hilly landform. The Jinxi River runs across the county. The main soil type in the study area is red soil. The main vegetation types are natural secondary forest, artificial fir, *Pinus massoniana* and *Phyllostachys pubescens*.

## Data collection and research methods

**Fig 2:**
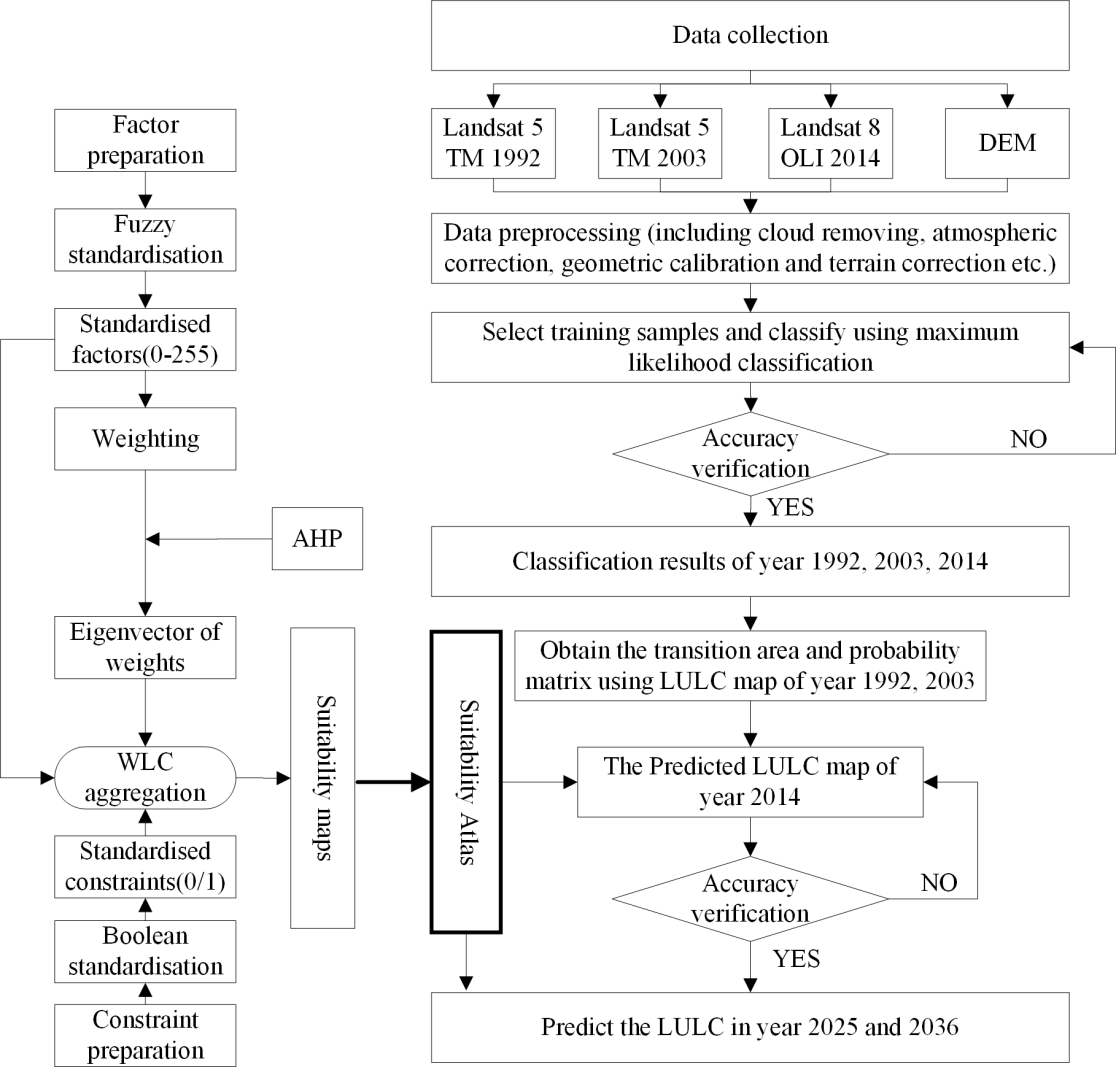
Data processing flow chart.

Fig 2 illustrates the framework of the prediction process. The major steps include (1) data preparation; (2) determination of the classification results of three years; and (3) the application of the CA-Markov model to obtain the predictions the LULCs in the years 2025 and 2036.

### Data collection

In this study, Landsat satellite remote sensing images from 1992, 2003 and 2014 are used, with a resolution of 30 m and track numbers of 120 / 41, 120 / 42. The detailed data are shown in Table 1. Documents such as the "Land use status classification" from the national standards and "Fujian Environmental Bulletin" and "Fujian Statistical Yearbook" are used.

**Table 1:** Data collection table.

Dataset	Date	Azimuth	Elevation angle
Landsat 8 OLI* (Path/Row:120/41,120/42)	Oct. 17, 2014	150.64	49.18
		149.44	50.31
Landsat 5 TM* (Path/Row:120/41,120/42)	Oct. 19, 2003	144.13	45.88
		146.92	46.89
Landsat 5 TM* (path/Row:12041,120/42)	Oct. 20, 1992	139.90	43.13
		138.71	44.07
ASTER GDEM	These data with 30-m spatial resolution were registered into the same coordinate system as the images and are used to conduct topographic correction of Landsat imagery.		
Topographic map	1: 50,000 Topographic map of Jiangle County		
Forest Management Inventory data of Jiangle County obtained in 2013, Google Earth image			
The shape files of streets and water in Jiangle were obtained from the Google Earth and OpenStreetMap(https://www.openstreetmap.org/).			

*: These data were collected from the official website of US Geological Survey (USGS) (http://glovis.usgs.gov).

### Remote sensing image preprocessing and accuracy verification

The remote sensing image data of the years 1992, 2003 and 2014 were radially calibrated and atmospherically corrected. The relative geometric corrections of the three images were conducted to remove geometric distortion caused by the sensor or the Earth rotation. Due to the differences between the TM and OLI sensors, the geometric correction of the year 2002 was based on the DEM data of the study area. Then, the 1992 and 2014 images were georeferenced to the 2002 image[48]. The errors were less than 1 pixel. Finally, terrain correction and image stitching were conducted. Considering the "China Land Classification System" and the goal of this study, the land use types were divided into five categories: farmland (including dry land and paddy field), woodland (the forest area), water, construction land (including settlements and roads) and bare land. Based on Google Earth images, Forest Management Inventory data and Landsat data of different periods, the training samples and the validation data of different periods were selected. With the help of the maximum likelihood method, classification was carried out on these three images. Precision testing was conducted using the Kappa index and the overall accuracy for the classification[53, 54]. Image processing was based on the UTM WGS 1984 (50N) projection system. The software ENVI 5.1 and ArcGIS 10.2 were used.

### Prediction of future LULC dynamics

#### Markov chain analysis

Markov Chain Analysis is frequently used to simulate complex processes such as land use change. It is mainly used to study the transition probability between an initial state and a final state to determine the transition trend among different land use states. Markov chain analysis is a random process that is discrete in both time and state[55–57]. The model simulation process mainly produces a land use area transfer matrix and a probability transfer matrix to predict land use change trends. Here, the Markov chain model could be described as a set of states, S = {S_0_, S_1_, S_2_, …, S_n_}, assuming that the current state is S_t_, and then, it changes to state S_j_ at the next step with a probability denoted by transition probabilities p_ij_. Thus, state S_t+1_ in the system could be determined by former stage S_t_ in the Markov chain using the following formula[58, 59]:

$$\begin{array}{l} P_{ij}=\left[\begin{array}{ccc} p_{11} & \cdots & p_{1n}\\ \vdots & \vdots & \vdots \\ p_{n1} & \cdots & p_{nn} \end{array}\right]\\ \left(0\leq P_{ij}<1\;and\;\sum_{j=1}^{n}P_{ij}=1,i,j=1,2,\cdots ,n\right) \end{array}$$ (1)

$$S_{t+1}=P_{i}{}_{j}\times S_{t}$$ (2)

where *P*_*ij*_ is the state transition probability matrix and n is the land use type number; S is land use status, t; t+1 is the time point. In this study, the Markov chain analysis was implemented in two periods: 1992 to 2003 and 2003 to 2014. Thus, the land use area transfer matrix and transition probability matrix were obtained.

#### Cellular automata model

Cellular Automata (CA) is a bottom-up dynamic model with a spatiotemporal calculation. It is discrete in space-time and state and can carry out complex time-space simulations[43]. The data for every cell in state S_t+1_ are decided by the cell itself and its neighboring cells in state S_t_, meaning that the change in the cell is decided by rules. The Cellular Automata model consists mainly of cell, cell space, neighbor, rule and time. The neighbors are determined by the filter of the CA model. The closer the distance between the nuclear cell and neighbor, the larger the weight factor will be. The weight factor is combined with the probabilities of transition to predict the state of adjacent grid cells, so that land use change is not a completely random decision. The commonly used neighborhoods are *Moore*, the *extended Moore*, and *von Neumann*. In this study, we used the *von Neumann* neighborhood. The rules are the suitability maps that show the possibility of the cell changing from one status to another. The model expression is:

$$S_{t+1}=f(S_{t},N)$$ (3)

where S is the set of states of the finite cells. The t and t+1 are different moments; N is the neighborhood of cells; and *f* is the transformation rule of local space.

#### CA-Markov model

There are no spatial variables in the Markov model, while the status for cells in the CA model is closely related to the spatial variables. The CA-Markov model integrates the CA model's ability to simulate the spatial variation in complex systems and the advantages of the long-term predictions of the Markov model. The Markov chain model component controls the temporal dynamics among the LULC classes based on transition probabilities, while the spatial dynamics are controlled by local rules determined either by the CA spatial filter or transition potential maps. The transition probabilities matrix produced by the Markov chain model is one of the inputs of the CA model[23]. The CA Markov model effectively combines the advantages of the Markov model and the CA model. The spatial prediction accuracy can be effectively simulated at the same time[60]. The process of prediction with the CA-Markov model is 1) building the suitability atlas based on the MCE, 2) generating the transfer matrix and the state transition probability matrix using the Markov model, and 3) predicting the future LULC using the CA model.

#### Suitability maps preparation

To use Cellular Automata, a suitability atlas for all the classes is considered as a prerequirement[17]. The suitability atlas contains a series of suitability maps, which are usually built through the multi-criteria evaluation (MCE). The basic point of MCE is to integrate different rules to derive a single index of evaluation[61]. The MCE includes two parts: the constraints (the hard rules) and the factors (the soft rules). The constraints are the criteria that limit the expansion of classes. The constraints are expressed in the form of Boolean maps where the areas that are not suitable will be set a value of 0, while the suitable areas will be set a value of 1. The factors give a degree of suitability for an area to change (mostly on distance basis) [62]. The process of data preparation is outlined in Fig 2. There are 3 steps: (1) Identification and development of the criteria, (2) standardization of the criteria and (3) aggregation of the criteria to obtain the suitability map for each class.

**Fig 3:**
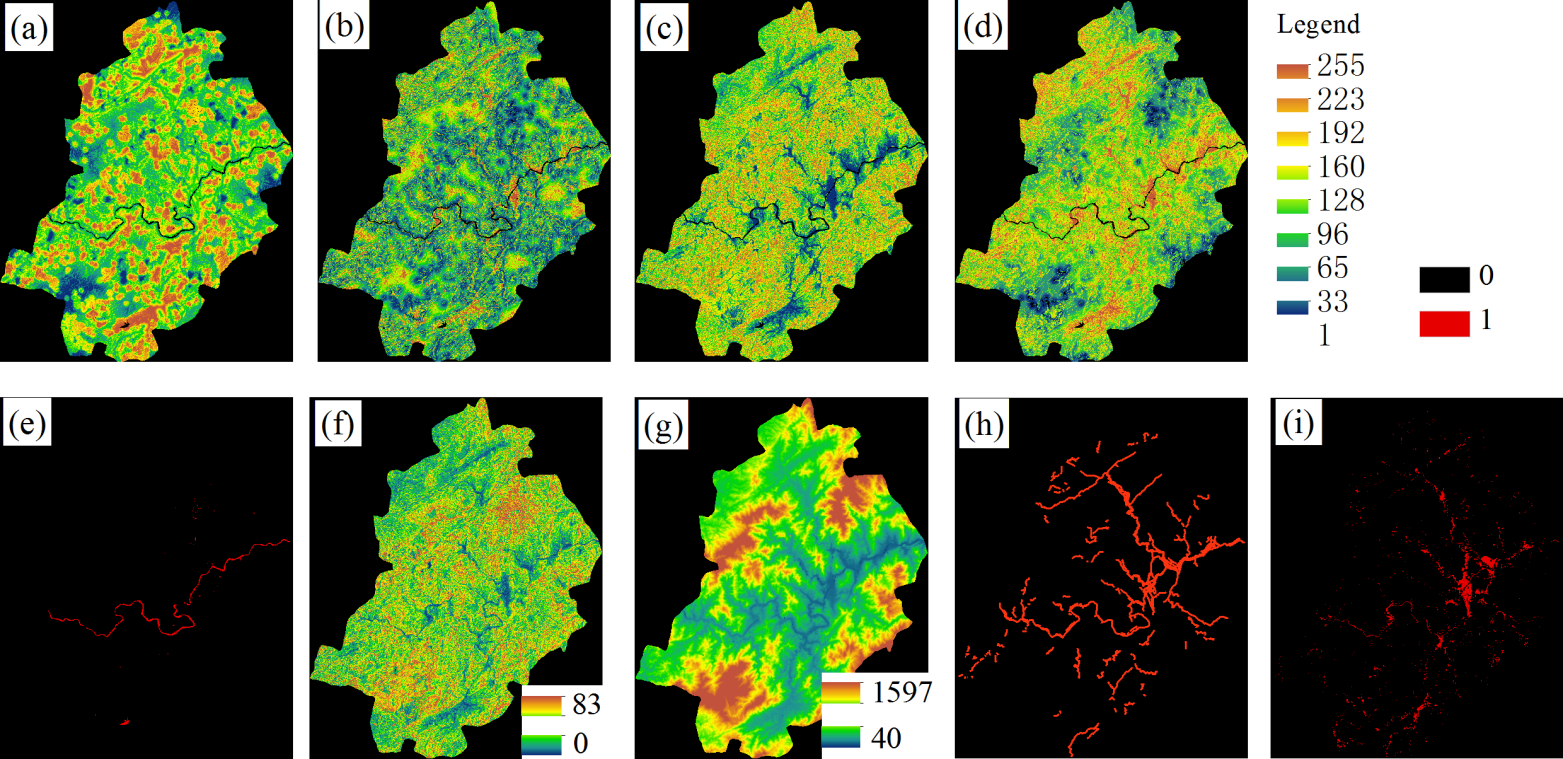
The input data and the suitability maps. Construction (b), Bare land (c), Woodland (d), Farmland(e) and Water (a) are suitability maps. Slope (f), Elevation (g), Road (h) and Construction (i) are the input data.

Due to the complexity of the terrain, social development and the Soil and Water Conservation Work Regulations, the existing slope, road, construction area, water are adopted to build the rules. The water and construction area were derived from the LULC maps. The road was downloaded from the OpenStreetMap and was checked according to the image data of each year. The slope was derived from the DEM data of study area with a resolution of 30×30 m. Factor and constraint images were first prepared in ArcGIS 10.0. Then, the images were imported into the IDRISI 17.0 for further processing. Using the Decision Wizard module in IDRISI, the suitability maps (Water, Construction, Bare land, Woodland and Farm land) were derived (Fig 3). First, the constraints for the year 2014 were standardized into Boolean maps. Here, we utilized two constraints, the water and existing construction, because no changes can take place in the waterbodies and existing construction areas. Second, the Fuzzy function combined with the Weighted Linear Combination (WLC) was used to process the standard factors. During the standardization, the factors were stretched from 0 to 255 using different fuzzy functions and control points. For different type of factors, the fuzzy functions can be Sigmoidal, J-shaped and Linear, with monotonically increasing/decreasing or symmetric. For the control points, we set them according to the statistical results or regulations from the government. The weights of the factors were derived with the AHP function in the WLC module. Third, the suitability map of a certain class was processed in the MCE module with the constraints, factors, and weights. Finally, the suitability atlas was obtained using Collection Editor in IDRISI.

#### LULC change prediction

In this study, the cell is the image grid cell, the unit size is 30×30 mm, and the whole land use spatial pattern is the cell space. The interval time is 11 years, so the number of cycles for the cellular automaton is 11. First, the land use transfer matrix and state transition probability matrix from 1992 to 2003 and 2003 to 2014 are calculated using the Markov module of IDRISI 17.0. Based on the MCE suitability map, the multi-objective decision-making module (multicriteria evaluation, MCE) in IDRISI is used to determine the suitability[63]. After comparing the results derived under the 3×3 filter with the 5×5 filter in CA model process, we adopted the 5×5 filter, meaning that the change in status for a central cell will be affected by the 5×5 neighbor cells. Finally, the land use predictions for 2025 and 2036 based on the data in 2003 and 2014 were carried out using the CA-Markov module integrated in IDRISI.

#### CA-Markov model validation

Model calibration and validation is an important step in the process of model prediction. The usefulness of a model depends on the output of the validation model. The Kappa index is one of the most popular used methods for quantifying the predictive power of a model[23]. That is to compare the predicted data with reference data using the VALIDATE module. Using the CROSSTAB module in IDRISI, the predicted LULC of 2014 is compared with the 2014 observed results to obtain the Kappa index. When the Kappa index is acceptable, the land use and land cover in 2025 and 2036 can be predicted.

### Analysis on dynamic change rate of land use

The rate of land use change reflects the severity of land use change in the study area in a given time period. The standard of measurement is divided into a single land use dynamic degree and a comprehensive land use dynamic degree[64–66]. In this study, we adopt a single land use dynamic degree and a comprehensive land use dynamic degree. In addition, a spatial analysis model of land use dynamic change on the basis of the dynamic degree proposed by Liu Shenghe and Shu Jin is also used to compare the differences between these two dynamic evaluation methods[67]. The formulas are as follows:

The single land use dynamic degree:

$$S_{i}=\frac{LA_{\left(i,t1\right)}-ULA_{i}}{LA_{\left(i,t1\right)}}\times \frac{1}{t_{2}-t_{1}}\times 100\%$$ (4)

The comprehensive land use dynamic degree:

$$S=\frac{\sum_{i=1}^{n}\left\{LA_{\left(i,t1\right)}-ULA_{i}\right\}}{\sum_{i=1}^{n}LA_{\left(i,t1\right)}}\times \frac{1}{t_{2}-t_{1}}\times 100\%$$ (5)

The spatial-based land use dynamic degree (the rate of change):

$$CCL_{i}=TRL_{i}+IRL_{i}$$ (6)

$$TRL_{i}=\frac{LA_{\left(i,t1\right)}-ULA_{i}}{LA_{\left(i,t1\right)}}\times \frac{1}{t_{2}-t_{1}}\times 100\%$$ (7)

$$IRL_{i}=\frac{LA_{\left(i,t2\right)}-ULA_{i}}{LA_{\left(i,t1\right)}}\times \frac{1}{t_{2}-t_{1}}\times 100\%$$ (8)

where *S*_*i*,_ is the dynamic degree of a single land use type and *S* is the comprehensive land use dynamic degree. *LA*_*(i*, *t1)*_ is the area of a certain type of land use at an earlier date, while *LA*_*(i*, *t2)*_ is the area of a certain type of land use at a later date. *ULA*_*i*_ is the part that is not changed. *t*_*1*_ and *t*_*2*_ represent the year before and after the change, respectively. *TRL*_*i*_ is the transfer-out rate, *IRL*_*i*_ is the transfer-in rate, and CCL_i_ is the sum of *TRL*_*i*_ and *IRL*_*i*_.

## Results and discussion

### Results of classification and analysis

**Fig 4:**
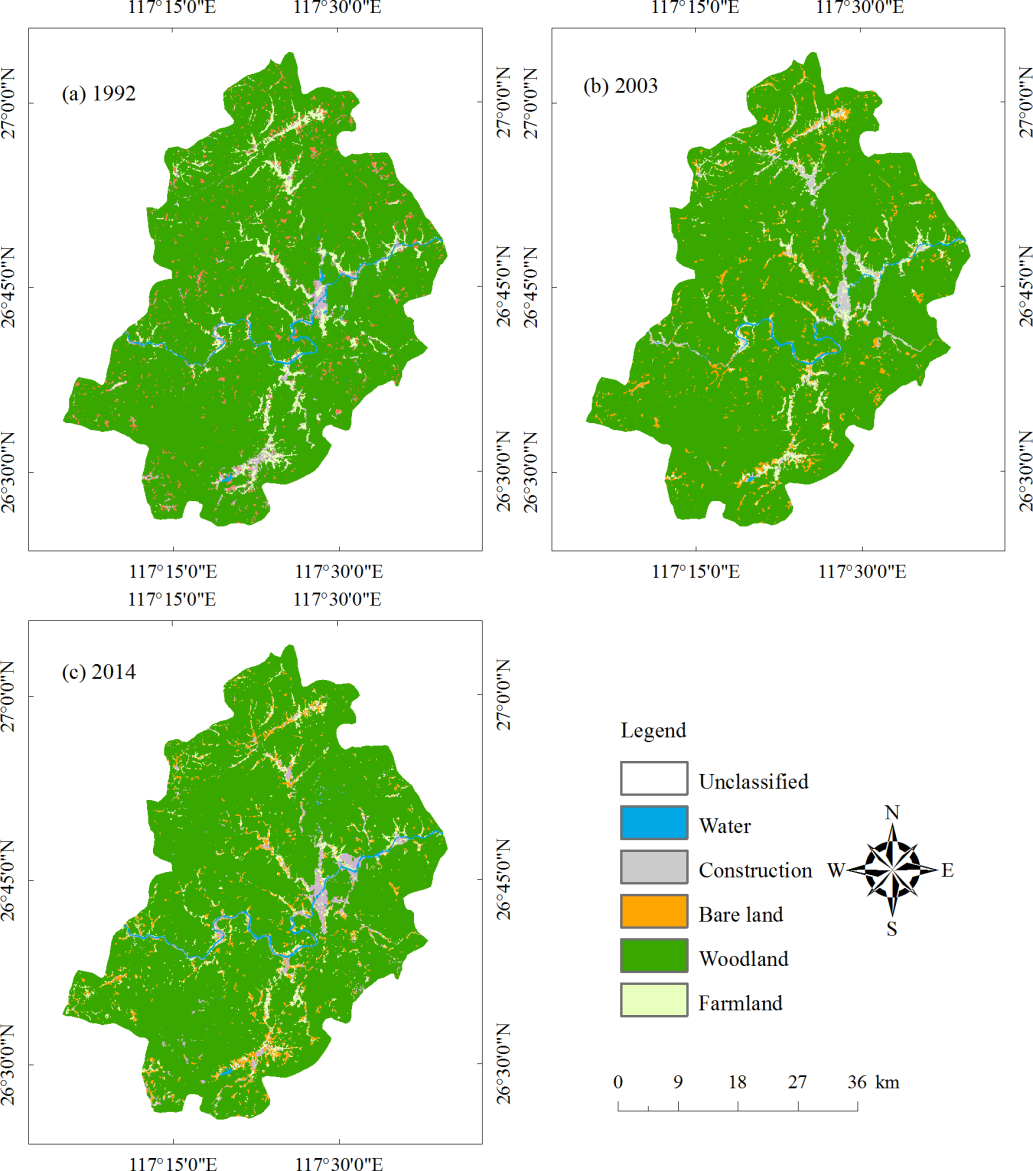
The classification map.

Classification results of the preprocessed images in 1992, 2003 and 2014 are presented in Fig 4. According to the classification results, the statistics for the three years of different types of land use areas and their proportions are shown in Table 2. It can be drawn from Table 2 and Fig 4 that the area of woodland is the largest in the study area. The woodland area are 2012.78 km^2^, 2020.76 km^2,^ and 1997.88 km^2^ in 1992, 2003 and 2014, respectively. Construction land increases gradually during the 22 years from 1992 to 2014, which is characteristic of the Chinese urbanization process. The water area shows a trend of decreasing first and then increasing. According to the images of the years and relevant data, water bodies were developed and dredged. Substantial sand excavation equipment existed in the early stage and sediment built up. The river width was narrower in 2014 than 1992, so the water area decreased sharply. Woodland and bare land changes correlate with urban expansion and wood cutting. The Jiangle state-owned forest farm is dominated by Chinese Fir, which is cut and planted for a fixed period.

**Table 2:** Area statistics for 1992, 2003 and 2014.

LULC Classes	1992		2003		2014	
	Area/km^2^	Area (%)	Area/km^2^	Area (%)	Area/km^2^	Area (%)
Water	20.18	0.89	13.87	0.61	20.15	0.89
Construction	54.01	2.39	63.22	2.80	69.08	3.06
Bare land	71.36	3.16	90.69	4.02	81.83	3.63
Woodland	2012.78	89.21	2020.76	89.56	1997.88	88.55
Farmland	97.94	4.34	67.74	3.00	87.34	3.87
Total	2256.28	100.00	2256.28	100.00	2256.28	100

In this study, 163 polygons for Landsat TM and 172 polygons for Landsat OLI are randomly selected to assess classification accuracy. The validation data are randomly and manually chosen based on Google Maps and the Forest Management Inventory. Table 3 contains the evaluation results of the three periods of images. Producer’s accuracy and user’s accuracy are obtained by a confusion matrix. Overall classification accuracy in 1992, 2003 and 2014 are 94.94%, 92.12% and 92.33%, respectively, with Kappa indexes of 0.9254, 0.8964 and 0.8746, respectively.

**Table 3:** Classification accuracy verification values.

LULC Classes	1992		2003		2014	
	Producer's accuracy (%)	User's accuracy (%)	Producer's accuracy (%)	User's accuracy (%)	Producer's accuracy (%)	User's accuracy (%)
Water	87.54	89.97	83.13	99.74	88.74	89.48
Construction	71.26	85.02	74.24	83.05	79.91	90.76
Bare land	90.47	88.01	93.48	83.54	93.81	79.92
Woodland	96.44	97.3	98.96	97.94	95.45	97.49
Farmland	97.24	83.8	97	67.69	89.55	59.86
Overall Accuracy (%)	92.05		92.54		91.33	
Kappa Coefficient	0.8808		0.8793		0.8625	

### Analysis of land use change

**Fig 5:**
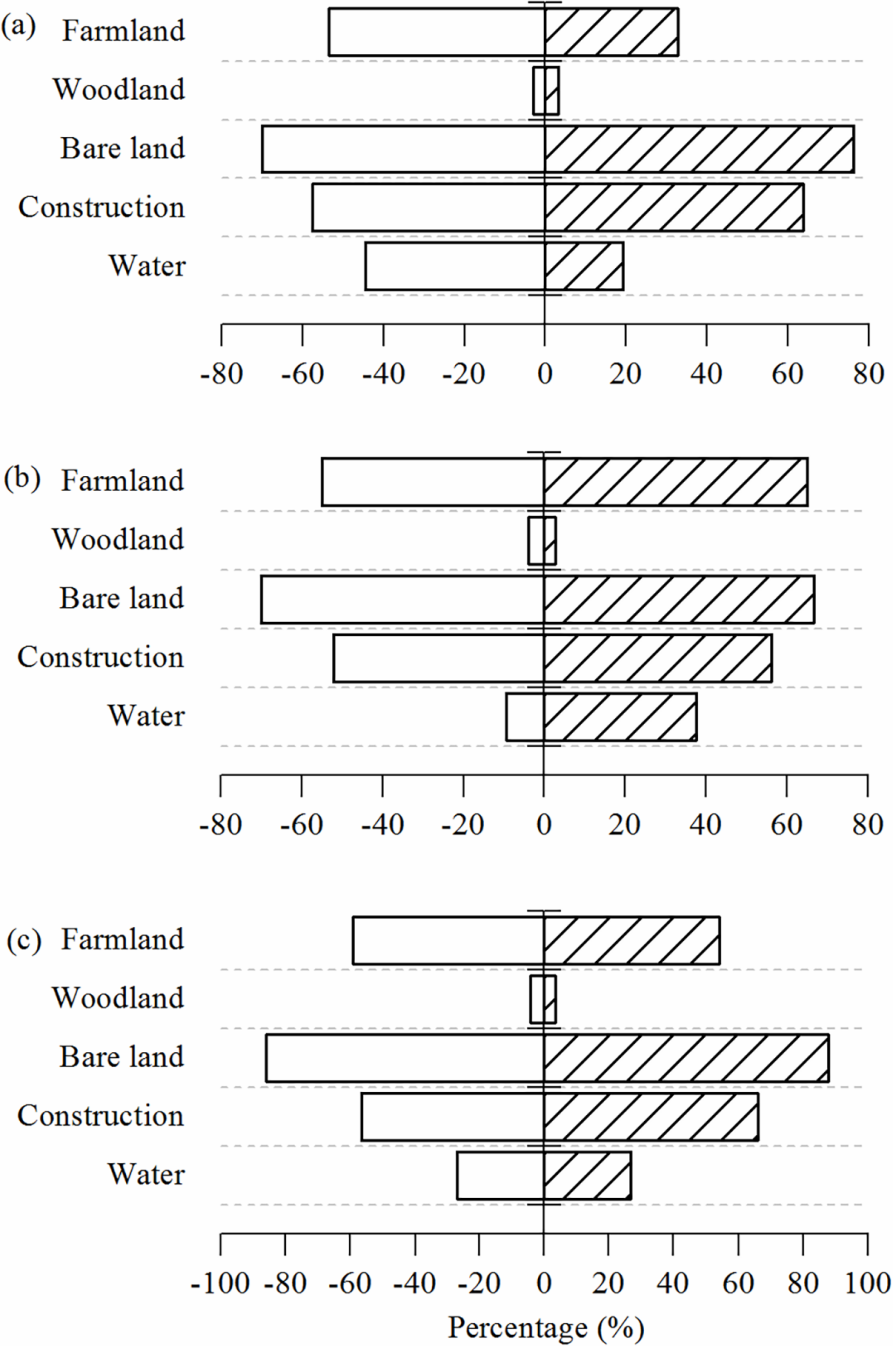
Comparison of land use increases and decreases from 1992–2003, 2003–2014, 1992–2014.

**Fig 6:**
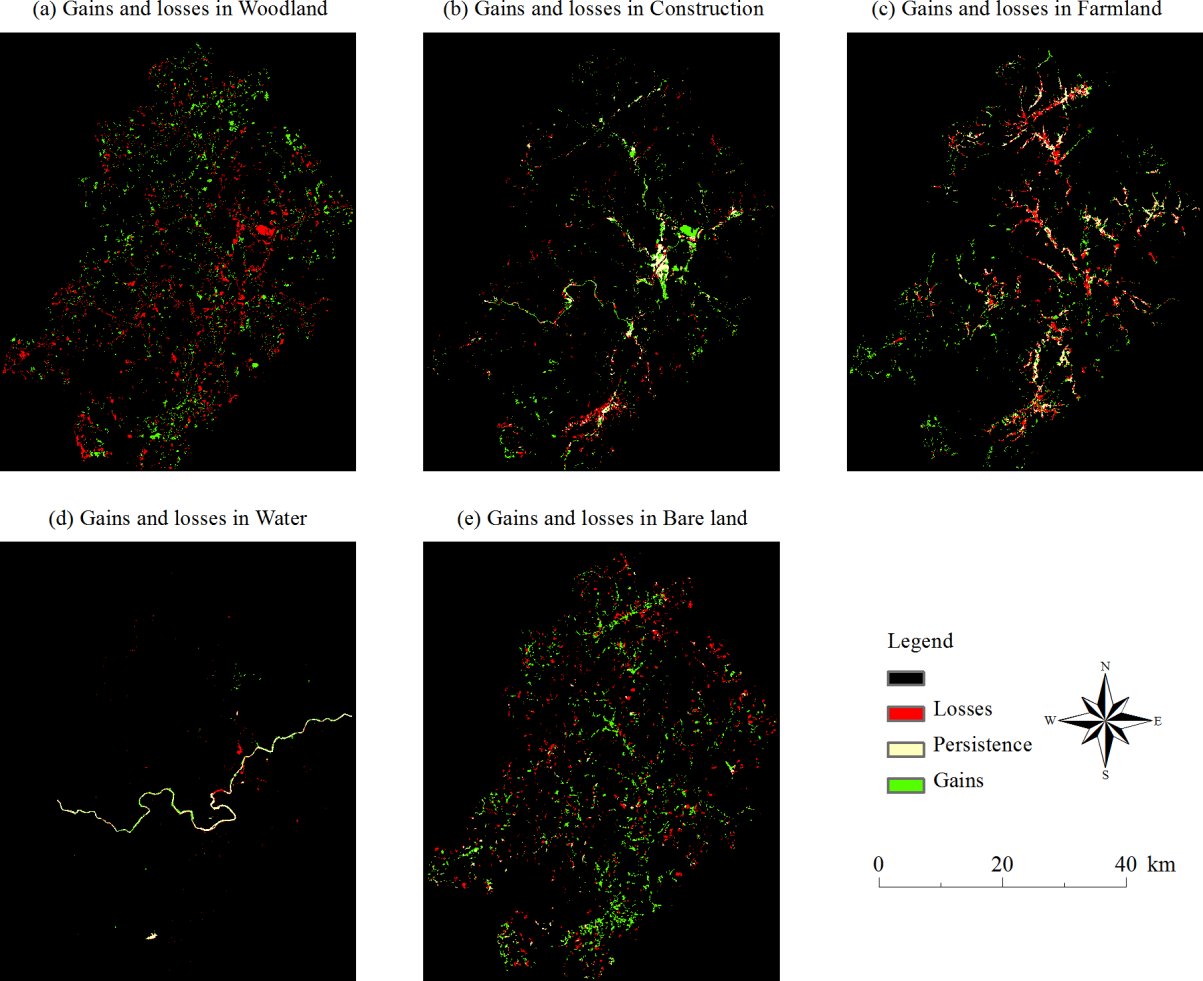
Map of the increases and decreases in different land use types from 1992 to 2014.

**Fig 7:**
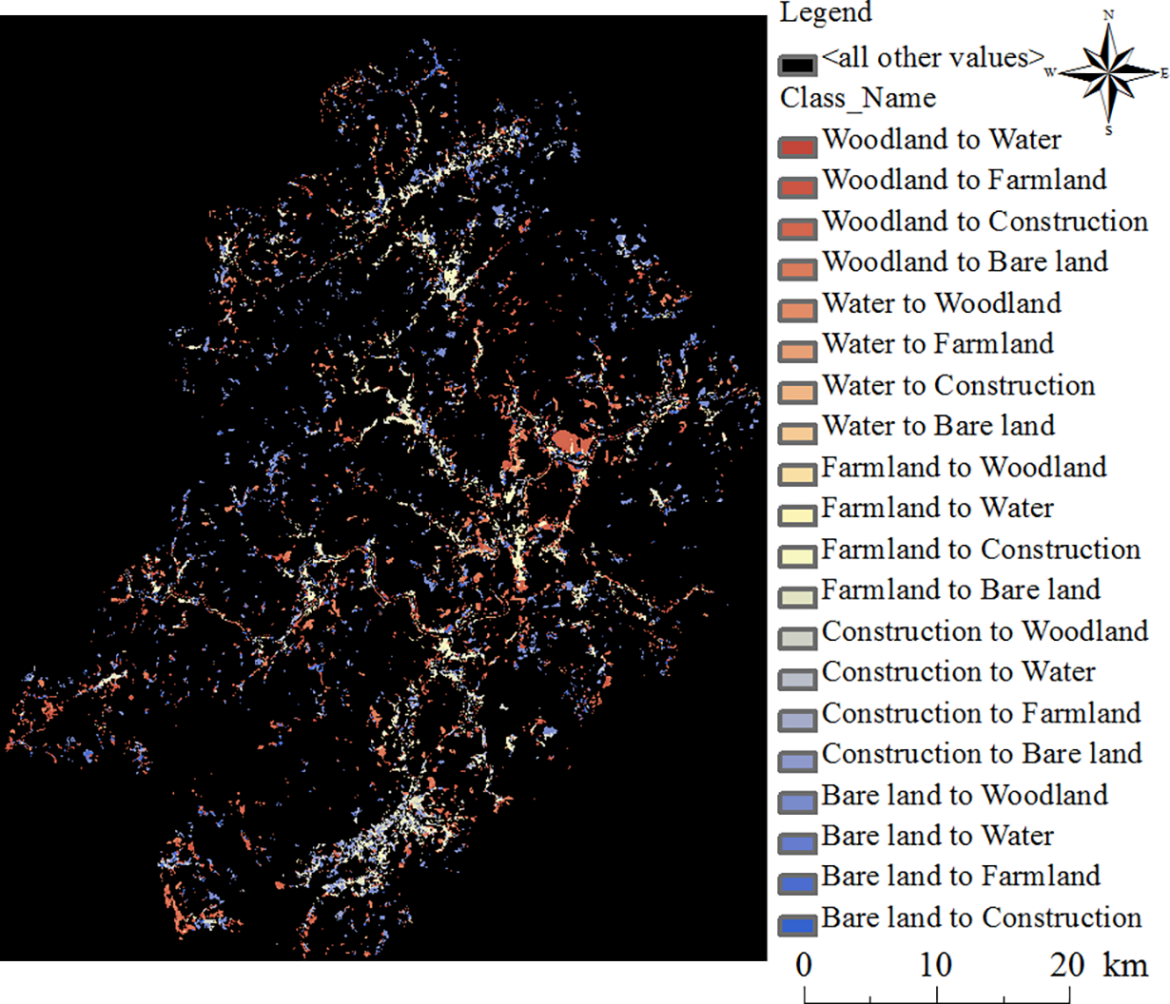
Changes in land use types from 1992 to 2014.

Fig 5 is the schematic diagram of each land use in the periods of 1992–2003 (Fig 5(A)), 2003–2014 (Fig 5(B)) and 1992–2014 (Fig 5(C)), and the diagrams show the increase and decrease of each land use. Table 4 is the statistical table of land use change in Jiangle. Considering Fig 5 and Table 4, it can be concluded that land use change is obvious in the three periods. Fig 6 is a sketch map of the increases and decreases in different land use types from 1992 to 2014. For a certain land use and land cover type, the green cells mean during that period, the LULC type of a cell is transferred from another LULC type into that specific LULC type. In contrast, the red cells mean that the certain land use type of LULC transferred out to other types. Fig 7 demonstrates the mutual transformation of different land use types from 1992 to 2014. In Fig 7, each color represents one kind of transformation.

**Table 4:** Table of changes in area from 1992 to 2003, 2003 to 2014, 1992 to 2014.

LULC Classes	1992–2003		2003–2014		1992–2014	
	Area/km^2^	Area (%)	Area/km^2^	Area (%)	Area/km^2^	Area (%)
Water	-6.32	-31.29	6.29	45.34	-0.03	-0.14
Construction	9.21	17.05	5.86	9.27	15.07	27.90
Bare land	19.33	27.08	-8.86	-9.77	10.47	14.67
Woodland	7.98	0.40	-22.88	-1.13	-14.91	-0.74
Farmland	-30.20	-30.83	19.60	28.93	-10.60	-10.82

From 1992–2003, the net changes in water area and farmland area are relatively large, at more than 30%. The amount of water area and farmland area that converts to other land use types are 6.32 km^2^ and 30.20 km^2^, accounting for 44.58% and 53.50%, respectively. The percent of other land use types that transfer into water and farmland are 19.35% and 32.77%, respectively. According to the remote sensing images and the material, sand excavation and mine exploration existed around Jinxi River, which flowed through Jiangle County; therefore, Jinxi River became narrower. Farmland transfers into construction land and bare land under the affection of urbanization process. According to the Table 4, construction land area has a net increase of 9.21 km^2^, accounting for 17.05%. In addition, the percent of this construction land that transfers to other land use types is 57.55%, and the amount of area that transfers into construction land is 63.74%. The net increase in bare land is 19.33 km^2^, accounting for 27.08% of the area, and the percent of area that transfers into and from bare land are 76.26% and 69.93%, respectively. The net increase in area of woodland is 7.98 km^2^, accounting for 0.4% of the area. The amount of area that transfers into and from woodland are 3.26% and 2.87%, respectively. Because of forest maturity and economy development, the increasing percent of the bare land area is relatively high in this period.

Of the area that changes during 2003 to 2014, water has the largest change, at 45.34%, with a net increase in area of 6.29 km^2^. The percent of area that transfers from water into other land use type is 9.42%, and the percent of area that transfers into water is 37.68%. According to Jinagle County annals, the water area increases sharply because of a series of improvements, sand cleaning, a decrease in sand excavation, etc., which were part of dredging the Jinxi River. The area of farmland increases by 28.93% compared with that of 2003, with a net area increase of 19.6 km^2^. The percent of area that transfers into farmland and from farmland are 65.08% and 55.08%, respectively. Construction land area increased slowly, with a net increase of 5.86 km^2^, accounting for 9.27%. The percent of area that transfers into and from construction land are 56.31% and 55.08%, respectively. The area of bare land and woodland both decrease. Bare land area decreases 8.86 km^2^, accounting for 9.77%. The percent of area that transfers into and from bare land area are 66.80% and 70.08%, respectively. Woodland area decreases 22.88 km^2^, accounting for 1.13%. The percent of area that transfers into and from woodland area are 2.72% and 3.81%, respectively.

According to the changes from 1992 to 2014, the water area is flat, and the changes from 1992 to 2003 and from 2003 to 2014 demonstrate dynamic changes due to improvements to the Jinxi River. Generally, the construction area increased from 54.01 km^2^ to 69.08 km^2^, accounting for 27.09% of the increasing area. The percent of the area that transfers into and from the construction are 66.06% and 56.57%, respectively. The bare land area increases from 71.36 km^2^ to 81.83 km^2^, accounting for 14.67%, and the percent of area that changed into and from bare land are 87.72% and 85.98%, respectively, and these changes are the result of economic development and forest cutting. The farmland area decreases from 97.94 km^2^ to 87.34 km^2^, accounting for 10.82% of the decreasing area. The percent of area that transfers into and from farmland area are 54.13% and 59.18%, respectively, and these changes are the comprehensive results of forest cutting and economic development. The percent of the woodland area changed little at 0.74%.

### Analysis of change rate between two different models

Table 5 and Table 6 are the statistical tables of the land use dynamic changes in Jiangle in 1992–2003 and 2003–2014. The fastest change rate from 1992 to 2003 is bare land, which is 15.17 km^2^.a^-1^, and its transfer rate and gain rate are 6.36 km^2^.a^-1^ and 8.82 km^2^.a^-1^, respectively. The bare land dynamic index is 6.36 km^2^.a^-1^. The second fastest change rate is construction land area, which is 12.01 km^2^.a^-1^. Construction land area transfer rate and gain rate are 5.23 km^2^.a^-1^ and 6.78 km^2^.a^-1^, respectively, and the construction land dynamic rate is 5.23 km^2^.a^-1^. Change rates for water and farmland are low, namely, 5.26 km^2^.a^-1^ and 6.92 km^2^.a^-1^, respectively. However, their dynamic rates are the highest, namely, 4.05 km^2^.a^-1^ and 4.86 km^2^.a^-1^, respectively. The change rate and dynamic rate of woodland are lowest, namely, 0.56 km^2^.a^-1^ and 0.26 km^2^.a^-1^, respectively. For the period of 2003–2014, farmland has the fastest change rate, which is 12.64 km^2^.a^-1^, and its transfer rate and gain rate are 5.01 km^2^.a^-1^ and 7.64 km^2^.a^-1^, respectively. The farmland dynamic rate is 5.01 km^2^.a^-1^. The next fastest change rate is for bare land, which is 11.86 km^2^.a^-1^. The transfer rate and gain rate of bare land are 6.37 km^2^.a^-1^ and 5.48 km^2^.a^-1^, respectively. The bare land dynamic rate is 6.37 km^2^.a^-1^. The construction land change rate is 10.34 km^2^.a^-1^, and its dynamic rate is 4.75 km^2^.a^-1^. The change rates of water and woodland are lower, which are 5.84 km^2^.a^-1^ and 0.59 km^2^.a^-1^, respectively. Their dynamic rates are 0.86 km^2^.a^-1^ and 0.35 km^2^.a^-1^, respectively.

The following conclusions can be drawn: the rate of change is larger than the dynamic rate. During these two periods, the transfer rate, gain rate, rate of change and the dynamic rate are all relatively large, meaning that the LULC change is intense. However, the rate of change is significantly larger than dynamic rate, so the single land use dynamic rate cannot properly describe the dynamic changes of the LULCC. For the whole area, the dynamic rate is the same as the rate of change.

**Table 5:** The rate of land use change in Jiangle County during 1992–2003.

LULC Classes	Unchanged area	Transfer		Gain		Rate of change /(km^2^.a^-1^)	Dynamic degree/%
		Area /km^2^	Transfer rate /(km^2^.a^-1^)	Area /km^2^	Gain rate /(km^2^.a^-1^)		
Water	11.18	9.00	4.05	2.68	1.21	5.26	4.05
Construction	22.93	31.09	5.23	40.29	6.78	12.01	5.23
Bare land	21.47	49.89	6.36	69.22	8.82	15.17	6.36
Woodland	1954.81	57.97	0.26	65.95	0.30	0.56	0.26
Farmland	45.54	52.40	4.86	22.20	2.06	6.92	4.86
Total	2055.93	200.35	0.81	200.35	0.81	39.93	0.81

**Table 6:** The rate of land use change in Jiangle County during 2003–2014.

LULC Classes	Unchanged area	Transfer		Gain		Rate of change /(km^2^.a^-1^)	Dynamic degree /%
		Area /km^2^	Transfer rate /(km^2^.a^-1^)	Area /km^2^	Gain rate /(km^2^.a^-1^)		
Water	12.56	1.31	0.86	7.59	4.98	5.84	0.86
Construction	30.19	33.03	4.75	38.89	5.59	10.34	4.75
Bare land	27.12	63.56	6.37	54.70	5.48	11.86	6.37
Woodland	1943.52	77.23	0.35	54.35	0.24	0.59	0.35
Farmland	30.43	37.31	5.01	56.91	7.64	12.64	5.01
Total	2043.83	212.44	0.86	212.44	0.86	41.27	0.86

### Analysis of land use change matrices

Table 7 and Table 8 are the land use change matrix and transfer matrix, respectively. Markov’s transfer matrix reveals different types of transfer probabilities while quantitatively demonstrating the land use transfer process. The rows of the table signify the land use status and transferring out situation in the preliminary state t1 of land use change, while the columns of the table represent the land use status and transferring in situation in the final state. As is shown in the tables, the highest proportion of net increase in area is bare land, whose net increase area is 19.33 km^2^, and its net increase area accounts for 27.08%. The main reason for the increase is the transfer from woodland and farmland, and the amounts of their transfer areas are 35.51 km^2^ and 23.93 km^2^, respectively, with state transition probabilities of 0.1065 and 0.2761, respectively. The second highest proportion of net increase in area is construction, which accounts for 17.05% of the net increasing area, with a net increased area of 9.21 km^2^. The net increase area of construction land is mainly from woodland transfer-in and farmland transfer-in, and their transfer-in areas are 19.61 km^2^ and 12.99 km^2^, respectively, with state transition probabilities of 0.2263 and 0.0393, respectively. The woodland net increase area is 7.98 km^2^, and it has the lowest net increase percent at 0.40%. The woodland net increase area is mainly from bare land transfer-in, whose transfer-in area and transfer probability are 43.37 km^2^ and 0.6472, respectively. Water and farmland areas both decrease. Water area decreased 6.32 km^2^ and has a decrease proportion of 31.29%. Water mainly transfers into construction land, whose transfer-out area is 4.52 km^2^ with a probability of 0.0266. Farmland transfer-out area is 30.20 km^2^, accounting for 30.83%. Farmland mainly transfers into bare land and construction land, whose areas are 23.93 km^2^ and 19.6 km^2^, respectively, with state transition probabilities of 0.1065 and 0.0393, respectively.

**Table 7:** Area transfer matrix for 1992–2003 (km^2^).

LULC Classes		2003					
		Water	Construction	Bare land	Woodland	Farmland	Total
1992	Water	11.18	4.52	0.77	2.26	1.44	20.18
	Construction	1.21	22.93	9.01	11.81	9.06	54.01
	Bare land	0.15	3.17	21.47	43.37	3.19	71.36
	Woodland	0.96	12.99	35.51	1954.81	8.51	2012.78
	Farmland	0.36	19.61	23.93	8.50	45.54	97.94
	Total	13.87	63.22	90.69	2020.75	67.74	2256.28

**Table 8:** Probability matrix of land use transfer from 1992 to 2003 (×10^−2^).

LULC Classes		2003				
		Water	Construction	Bare land	Woodland	Farmland
1992	Water	47.11	26.6	4.53	13.31	8.46
	Construction	2.49	36.08	18.51	24.28	18.64
	Bare land	0.23	4.73	25.56	64.72	4.76
	Woodland	0.29	3.93	10.65	82.56	2.57
	Farmland	0.41	22.63	27.61	9.81	39.53

Table 9 and Table 10 are the land use change transfer matrix and transition probability matrix, respectively. The preliminary stage and the final state of changes are 2003 and 2014 with an 11-year interval. As can be seen from the table, water has the highest net area percent for the land use changes during 2003–2014. The net increase area is 6.29 km^2^, with a proportion of 45.34%. The net increase area mainly comes from construction, which is 3.77 km^2^, and the state transition probability is 0.0677. The second highest net area percent is farmland, which accounts for 29.93%. The farmland increase area is 19.60 km^2^, which mainly comes from woodland and bareland with transferring areas of 26.47 km^2^ and 18.27 km^2^, respectively, and with transition probabilities of 0.0628 and 0.2127, respectively. Construction land has a net increase proportion of 9.27% and its net increase area is 5.86 km^2^. Construction land increases mainly come from the transfer-in of woodland and farmland, and their transfer-in areas are 21.14 km^2^ and 10.51 km^2^, respectively, with state transition probabilities of 0.0501 and 0.1744, respectively. For the bare land, the net decrease proportion is 9.77%, with a net decrease of 8.86 km^2^. Bare land mainly transfers into woodland, farmland and construction land, with transfer-out areas of 38.57 km^2^, 18.27 km^2^ and 6.10 km^2^, respectively, and transition probabilities of 0.4538, 0.2127 and 0.0718, respectively. The woodland net area change proportion is the lowest, with a decrease proportion of 1.13%. However, woodland net area change amount is the highest, at 22.88 km^2^. Woodland mainly transfers into bare land, farmland and construction land, whose areas are 28.01, 26.47 and 21.14 km^2^, respectively. The corresponding transition probabilities are 0.0657, 0.0628 and 0.0501, respectively.

**Table 9:** Area transfer matrix for 2003–2014 (km^2^).

LULC Classes		2014					
		Water	Construction	Bare land	Woodland	Farmland	Total
2003	Water	12.56	1.14	0.02	0.07	0.08	13.87
	Construction	3.77	30.19	8.74	8.44	12.09	63.22
	Bare land	0.63	6.10	27.12	38.57	18.27	90.69
	Woodland	1.62	21.14	28.01	1943.52	26.47	2020.76
	Farmland	1.59	10.51	17.94	7.27	30.43	67.74
	Total	20.15	69.08	81.83	1997.88	87.34	2256.28

**Table 10:** Probability matrix of land use transfer from 2003 to 2014 (×10^−2^).

LULC Classes		2014				
		Water	Construction	Bare land	Woodland	Farmland
2003	Water	76.99	20.02	0.29	1.27	1.44
	Construction	6.77	40.59	15.71	15.18	21.75
	Bare land	0.74	7.18	25.44	45.38	21.27
	Woodland	0.38	5.01	6.57	81.76	6.28
	Farmland	2.63	17.44	29.71	12.04	38.18

### Results validation (observed LULC of 2014, simulated LULC of 2014)

**Fig 8:**
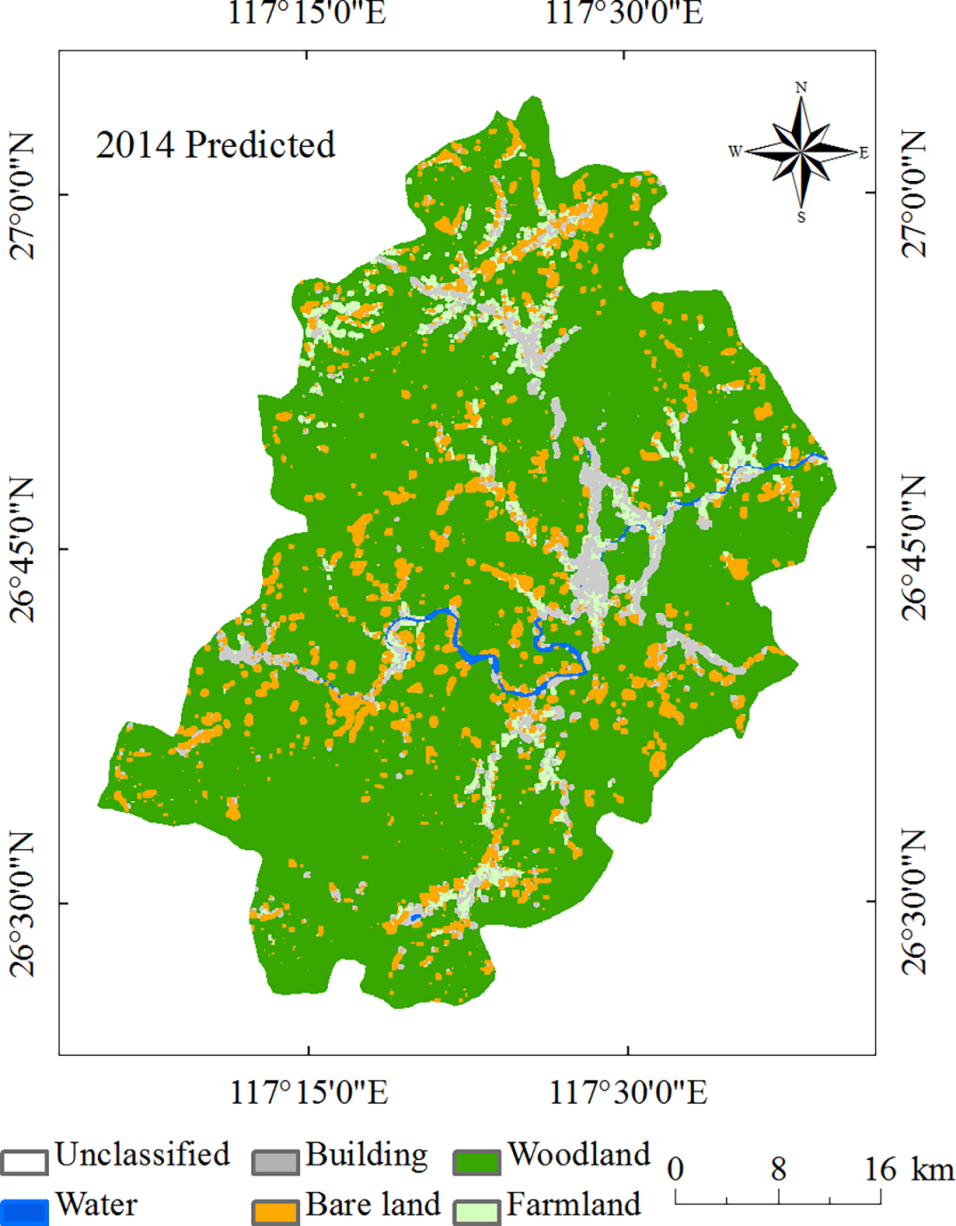
The predicted land use classification of Jiangle County in 2014.

The state transition area matrix and state transition probability matrix are created according to land use maps in 1992 and 2003, which can be obtained by running the CA-Markov model in IDRISI software based on the suitability atlas that has already been created. The predictive results map for 2014 is obtained with a 5×5 contiguity filter, whose running cycle is 11 years. Fig 8 is the predicted map of 2014. The next step is to use the CROSSTAB module in IDRISI. Predictive results are analyzed by overlaying the land use map of 2014 that is truly classified. Commonly, if the Kappa index is less than or equal to 0.4, then the land uses changed greatly and with poor consistency between the two images. If the Kappa index is 0.4–0.75, then there are general consistencies and obvious changes between the two images. Otherwise, there is high consistency between two images[58]. Usually, the Kappa values range from 0 to 1. Values of 0.61–0.80 means substantial, while 0.81–1 means almost perfect[68, 69]. Here, the Kappa index between the predicted map and the observed map of 2014 is 0.8128, which is above 0.75, illustrating that the results are reliable. There is high consistency between the actual observed results and predictive results. The precision for correct predictions is relatively high; therefore, this method can be used to predict the results in 2025 and 2036.

### Results prediction

**Fig 9:**
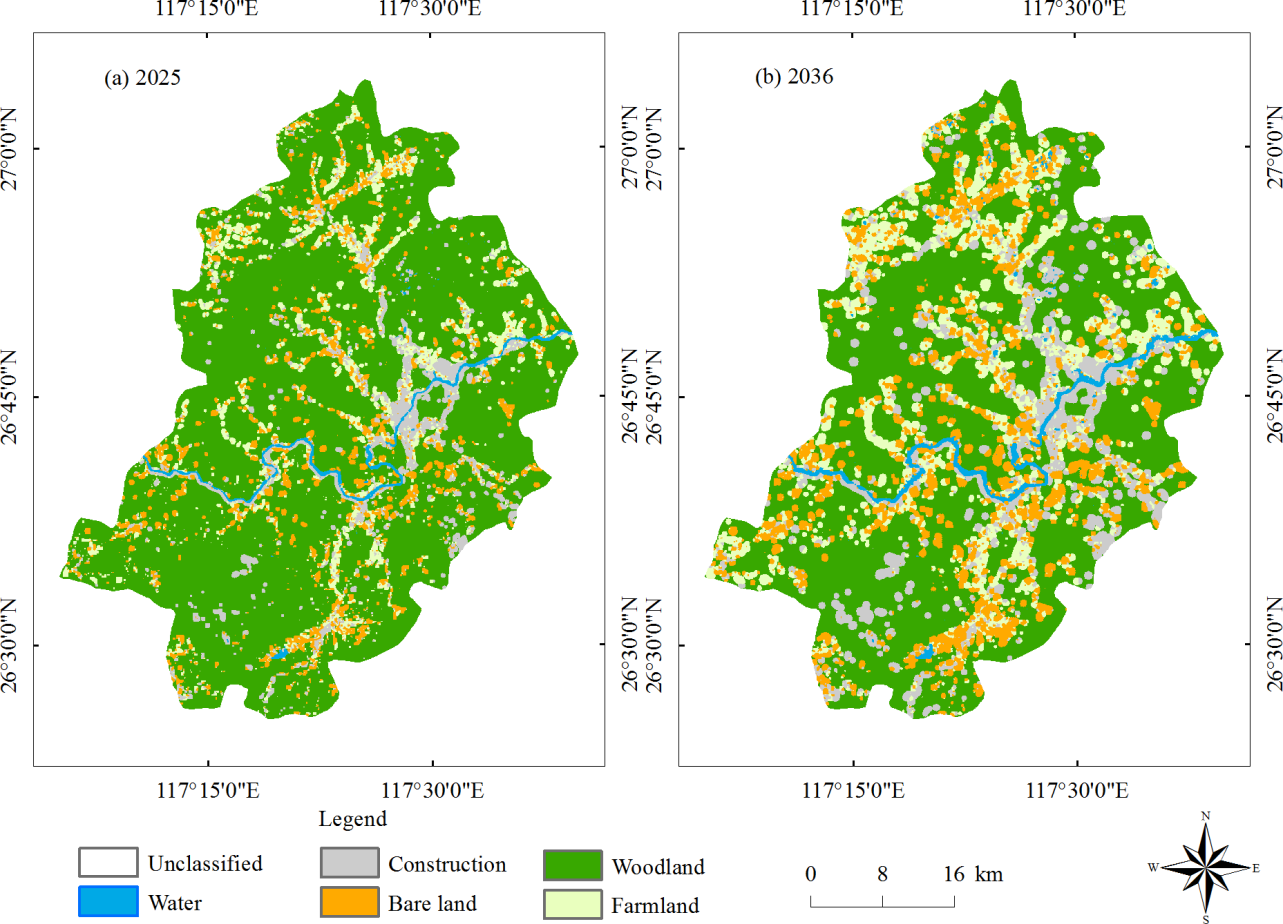
Predicted results of 2025 (left) and 2036 (right).

The state transition area matrix and state transition probability matrix are created by land use maps for 2003 and 2014, and the results for 2025 and 2036 are predicted by the same method (Fig 9). For the prediction of 2025, the interval time is 11 years, while for the year of 2036, the interval time is 22 years. Table 11 is the statistical table based on the predictive results in 2025 and 2036. In general, in 2025 and 2036, the woodland area decreased greatly, and the remaining land area increased by a certain amount, especially in 2036, and the area of woodland decreased significantly. Therefore, to consider ecology, the protection of woodland is necessary in planning.

**Table 11:** Statistics for 2025 and 2036.

LULC Classes	2025		2036	
	Area/km^2^	Area (%)	Area/km^2^	Area (%)
Water	26.81	1.19	48.67	2.16
Construction	153.27	6.79	268.71	11.91
Bare land	188.81	8.37	341.03	15.11
Woodland	1696.30	75.17	1255.73	55.65
Farmland	191.32	8.48	342.38	15.17
Total	2256.52	100.00	2256.52	100.00

## Conclusion

In recent years, with economic development and the impact of human activities, the county's land use has experienced substantial changes since the 1990s. In this study, Landsat5 TM and Landsat8 OLI image data were used to obtain land use maps for 1992, 2003 and 2014. Then, the land use structure of the study area was simulated and predicted based on the CA-Markov model.

According to the results of the classification, the forest coverage rate of Jiangle County was high, and the forest areas in 1992, 2003 and 2014 were 2012.78, 2020.76 and 977.88 km^2^, respectively. Construction land increased from 1992 to 2014 year by year. Water, bare land, and farmland area changes were closely related to human activities.

Under the influence of human activities, the land use changes in Jiangle County from 1992 to 2014 were obvious. The water area decreased first and then increased. In 1992–2003, a large amount of sand mining equipment was built in the Jinxi River, and a large amount of sediment was deposited on the river bank, so that the water area was drastically reduced. In 2003–2014, river sand mining equipment had reduced significantly, and the river was cleared, which led to the gradual restoration of the water area. The woodland area is large in size, although the changed area is large, and the proportion is small. Changes in the woodland area are mainly related to timber harvesting and urban expansion. The results showed that in 2025 and 2036, the area of woodland decreased drastically. Taking into account of the ecological functions of woodland, we should pay attention to the amount of woodland harvest in planning.

In the experimental process, there were some points that affected the prediction results. First, there were some difficulties in data acquisition due to the location of the study area. In addition, the study area is in the hilly area, where the ground changes in altitude, which has a certain impact on the image pixel value and ultimately leads to inaccuracy of the classification results. Second, the setting of the suitability data set had some influence on the LULC predictions. Finally, there was some human impacts on the land use types, especially the construction land changes. Therefore, through improving the quality of the input data and the setting of related parameters, the accuracy of the predicted LULC scenarios can be increased.

## References

[1] Han H, Yang C, Song J. Scenario Simulation and the Prediction of Land Use and Land Cover Change in Beijing, China. Sustainability-Basel. 2015;7(4):4260–79. 10.3390/su7044260

[2] Rawat JS, Kumar M. Monitoring land use/cover change using remote sensing and GIS techniques: A case study of Hawalbagh block, district Almora, Uttarakhand, India. The Egyptian Journal of Remote Sensing and Space Science. 2015;18(1):77–84. 10.1016/j.ejrs.2015.02.002.

[3] McConnell WJ. Land Change: The Merger of Land Cover and Land use Dynamics A2—Wright, James D International Encyclopedia of the Social & Behavioral Sciences (Second Edition). Oxford: Elsevier; 2015 p. 220–3.

[4] Lambin E. Land Cover Assessment and Monitoring. Encyclopedia of Analytical Chemistry: John Wiley & Sons, Ltd; 2006.

[5] Arsanjani JJ. Dynamic Land Use / Cover Change Modelling : Geosimulation and Agent-Based Modelling Vienna: University of Vienna; 2011.

[6] Li L, Lu D, Kuang W. Examining Urban Impervious Surface Distribution and Its Dynamic Change in Hangzhou Metropolis. Remote Sens-Basel. 2016;8(3). 10.3390/rs8030265

[7] Basommi LP, Guan Q-f, Cheng D-d, Singh SK. Dynamics of land use change in a mining area: a case study of Nadowli District, Ghana. J Mt Sci. 2016;13(4):633–42. 10.1007/s11629-015-3706-4

[8] Li X, Wang Y, Li J, Lei B. Physical and Socioeconomic Driving Forces of Land-Use and Land-Cover Changes: A Case Study of Wuhan City, China. Discrete Dyn Nat Soc. 2016 10.1155/2016/8061069

[9] Pielke RA, Sr., Pitman A, Niyogi D, Mahmood R, McAlpine C, Hossain F, et al Land use/land cover changes and climate: modeling analysis and observational evidence. Wires Clim Change. 2011;2(6):828–50. 10.1002/wcc.144

[10] Verburg PH, Eck JRRv, Nijs TCMd, Dijst MJ, Schot P. Determinants of Land-Use Change Patterns in the Netherlands. Environ Plann B. 2004;31(1):125–50. 10.1068/b307

[11] Behera MD, Borate SN, Panda SN, Behera PR, Roy PS. Modelling and analyzing the watershed dynamics using Cellular Automata (CA)–Markov model–A geo-information based approach. J. Earth Syst. Sci.. 2012;121(4):1011–24. 10.1007/s12040-012-0207-5

[12] Yin J, Yin Z, Zhong H, Xu S, Hu X, Wang J, et al Monitoring urban expansion and land use/land cover changes of Shanghai metropolitan area during the transitional economy (1979–2009) in China. Environ Monit Assess. 2011;177(1–4):609–21. 10.1007/s10661-010-1660-8 20824336

[13] Caldas M, Walker R, Arima E, Perz S, Aldrich S, Simmons C. Theorizing Land Cover and Land Use Change: The Peasant Economy of Colonization in the Amazon Basin. Ann Assoc Am Geogr. 2007;97(1):86–110.

[14] Galicia L, Garcia-Romero A. Land use and land cover change in highland temperate forests in the Izta-Popo National Park, central Mexico. Mt Res Dev. 2007;27(1):48–57. 10.1659/0276-4741(2007)27[48:lualcc]2.0.co;2

[15] Scanlon BR, Reedy RC, Stonestrom DA, Prudic DE, Dennehy KF. Impact of land use and land cover change on groundwater recharge and quality in the southwestern US. Glob Change Biol. 2005;11(10):1577–93. 10.1111/j.1365-2486.2005.01026.x

[16] Fox J, Vogler JB. Land-use and land-cover change in montane mainland southeast Asia. Environmental Management. 2005;36(3):394–403. 10.1007/s00267-003-0288-7 16132447

[17] Hyandye C, Martz LW. A Markovian and cellular automata land-use change predictive model of the Usangu Catchment. Int J Remote Sens. 2017;38(1):64–81. 10.1080/01431161.2016.1259675

[18] Lu D, Mausel P, Brondízio E, Moran E. Change detection techniques. Int J Remote Sens. 2004;25(12):2365–401. 10.1080/0143116031000139863

[19] Reis S. Analyzing Land Use/Land Cover Changes Using Remote Sensing and GIS in Rize, North-East Turkey. Sensors. 2008;8(10):6188–202. 10.3390/s8106188 27873865 PMC3707445

[20] Pervez W, Uddin V, Khan SA, Khan JA. Satellite-based land use mapping: comparative analysis of Landsat-8, Advanced Land Imager, and big data Hyperion imagery. J Appl Remote Sens. 2016;10 10.1117/1.jrs.10.026004

[21] Srivastava PK, Singh SK, Gupta M, Thakur JK, Mukherjee S. Modeling Impact of Land Use Change Trajectories on Groundwater Quality Using Remote Sensing and GIS. Environ Eng Manag J. 2013;12(12):2343–55.

[22] Pradhan B, Lee S, Mansor S, Buchroithner M, Jamaluddin N, Khujaimah Z. Utilization of optical remote sensing data and geographic information system tools for regional landslide hazard analysis by using binomial logistic regression model. J Appl Remote Sens. 2008;2 10.1117/1.3026536

[23] Singh SK, Laari PB, Mustak S, Srivastava PK, Szabó S. Modelling of land use land cover change using earth observation data-sets of Tons River Basin, Madhya Pradesh, India. Geocarto Int. 2017:1–34.

[24] Hua AK. Land Use Land Cover Changes in Detection of Water Quality: A Study Based on Remote Sensing and Multivariate Statistics. Journal of environmental and public health. 2017;2017:7515130-. 10.1155/2017/7515130 28377790 PMC5362731

[25] Olokeogun OS, Iyiola K, Iyiola OF. Application of remote sensing and GIS in land use/land cover mapping and change detection in Shasha forest reserve, Nigeria. ISPRS—Int Arch Photogramm. 2014;XL-8(8):613–6.

[26] Rai PK, Vishwakarma CA, Thakur S, Kamal V, Mukherjee S. Changing Land Trajectories: A Case Study from India Using a Remote Sensing Based Approach. European Journal of Geography. 2016;7(2):63–73.

[27] Mishra VN, Rai PK, Kumar P, Prasad R. Evaluation of land use/land cover classification accuracy using multi-resolution remote sensing images. Forum geografic. 2016;XV(1):45–53. 10.5775/fg.2016.137.i

[28] Khan S, Qasim S, Ambreen R, Syed ZUH. Spatio-Temporal Analysis of Landuse/Landcover Change of District Pishin Using Satellite Imagery and GIS. Journal of Geographic Information System. 2016;8(3):361–8.

[29] Zeleke G, Hurni H. Implications of Land Use and Land Cover Dynamics for Mountain Resource Degradation in the Northwestern Ethiopian Highlands. Mt Res Dev. 2001;21(2):184–91. 10.1659/0276-4741(2001)021[0184:IOLUAL]2.0.CO;2

[30] Paegelow M, Olmedo MTC. Possibilities and limits of prospective GIS land cover modelling—a compared case study: Garrotxes (France) and Alta Alpujarra Granadina (Spain). Int J Geogr Inf Sci. 2005;19(6):697–722. 10.1080/13658810500076443

[31] Shamsi SRF. Integrating Linear Programming and Analytical Hierarchical Processing in Raster-GIS to Optimize Land Use Pattern at Watershed Level. Journal of Applied Sciences and Environmental Management. 2010;14(2):81–5.

[32] Hyandye C. GIS and Logit Regression Model Applications in Land Use/Land Cover Change and Distribution in Usangu Catchment. American Journal of Remote Sensing 2015;3(1):6–16.

[33] Aitkenhead MJ, Aalders IH. Predicting land cover using GIS, Bayesian and evolutionary algorithm methods. J Environ Manage. 2009;90(1):236–50. 10.1016/j.jenvman.2007.09.010 18079039

[34] Singh SK, Mustak S, Srivastava PK, Szabó S, Islam T. Predicting Spatial and Decadal LULC Changes Through Cellular Automata Markov Chain Models Using Earth Observation Datasets and Geo-information. Environmental Processes. 2015;2(1):61–78.

[35] Yang X, Zheng X-Q, Lv L-N. A spatiotemporal model of land use change based on ant colony optimization, Markov chain and cellular automata. Ecol Model. 2012;233:11–9. 10.1016/j.ecolmodel.2012.03.011

[36] Subedi P, Subedi K, Thapa B. Application of a Hybrid Cellular Automaton–Markov (CA-Markov) Model in Land-Use Change Prediction: A Case Study of Saddle Creek Drainage Basin, Florida. Science & Education. 2013;1(6):126–32.

[37] Stefanov WL, Ramsey MS, Christensen PR. Monitoring urban land cover change: An expert system approach to land cover classification of semiarid to arid urban centers. Remote Sens Environ. 2001;77(2):173–85.

[38] Ralha CG, Abreu CG, Coelho CGC, Zaghetto A, Macchiavello B, Machado RB. A multi-agent model system for land-use change simulation. Remote Sens Environ. 2013;42:30–46. 10.1016/j.envsoft.2012.12.003

[39] Sohl TL, Claggett PR. Clarity versus complexity: Land-use modeling as a practical tool for decision-makers. J Environ Manage. 2013;129:235–43. 10.1016/j.jenvman.2013.07.027. 10.1016/j.jenvman.2013.07.027 23954777

[40] Zhao L, Peng Z-R. LandSys: an agent-based Cellular Automata model of land use change developed for transportation analysis. J Transp Geogr. 2012;25:35–49. 10.1016/j.jtrangeo.2012.07.006.

[41] Stevens D, Dragićević S. A GIS-Based Irregular Cellular Automata Model of Land-Use Change. Environ Plann B 2007;34(4):708–24. 10.1068/b32098

[42] Myint SW, Wang L. Multicriteria decision approach for land use land cover change using Markov chain analysis and a cellular automata approach. Can J Remote Sens. 2006;32(6):390–404. 10.5589/m06-032

[43] He D, Zhou J, Gao W, Guo H, Yu S, Liu Y. An integrated CA-markov model for dynamic simulation of land use change in Lake Dianchi watershed. Beijing Daxue Xuebao (Ziran Kexue Ban)/Acta Scientiarum Naturalium Universitatis Pekinensis. 2014;50(6):1095–105.

[44] He C, Okada N, Zhang Q, Shi P, Zhang J. Modeling urban expansion scenarios by coupling cellular automata model and system dynamic model in Beijing, China. Appl Geogr. 2006;26(3):323–45. 10.1016/j.apgeog.2006.09.006.

[45] Wu C-D, Cheng C-C, Lo H-C, Chen Y-K. Application of SEBAL and Markov Models for Future Stream Flow Simulation Through Remote Sensing. Water Resour Manag. 2010;24(14):3773–97. 10.1007/s11269-010-9633-9

[46] Memarian H, Balasundram SK, Talib JB, Sung CTB, Sood AM, Abbaspour K. Validation of CA-Markov for Simulation of Land Use and Cover Change in the Langat Basin, Malaysia. Journal of Geographic Information System. 2012;4(6):542–54.

[47] Rendana M, Rahim SA, Mohd RIW, Lihan T, Rahman ZA. CA-Markov for predicting land use changes in tropical catchment area: a case study in Cameron Highland, Malaysia. Journal of Applied Sciences. 2015;15(4):689–95.

[48] Nguyen TA, Le PMT, Pham TM, Hoang HTT, Nguyen MQ, Ta HQ, et al Toward a sustainable city of tomorrow: a hybrid Markov–Cellular Automata modeling for urban landscape evolution in the Hanoi city (Vietnam) during 1990–2030. Environment, Development and Sustainability. 2017 10.1089/sus.2017.29092.aml

[49] Wu Y, Li S, Yu S. Monitoring urban expansion and its effects on land use and land cover changes in Guangzhou city, China. Environ Monit Assess. 2016;188(1). 10.1007/s10661-015-5069-2 26700678

[50] Li Y-y, Zhang H, Kainz W. Monitoring patterns of urban heat islands of the fast-growing Shanghai metropolis, China: Using time-series of Landsat TM/ETM+ data. Int J Appl Earth Obs. 2012;19:127–38. 10.1016/j.jag.2012.05.001

[51] Lin X, Gao J, Zhang Y. Superiority in and Development Path of the “Maritime Silk Road” Construction in Fujian Province. Marine Economy. 2014.

[52] Xv H, Sun Y, Wang X, Wang J, Fu Y. Linear mixed-effects models to describe individual tree crown width for china-fir in fujian province, southeast china. Plos One. 2015;10(4):e0122257 10.1371/journal.pone.0122257 25876178 PMC4398382

[53] Congalton RG. A review of assessing the accuracy of classifications of remotely sensed data. Remote Sens Environ. 1991;37(1):35–46. 10.1016/0034-4257(91)90048-B.

[54] Keshtkar H, Voigt W, Alizadeh E. Land-cover classification and analysis of change using machine-learning classifiers and multi-temporal remote sensing imagery. Arabian Journal of Geosciences. 2017;10(6). 10.1007/s12517-017-2899-y

[55] Mishra VN, Rai PK. A remote sensing aided multi-layer perceptron-Markov chain analysis for land use and land cover change prediction in Patna district (Bihar), India. Arabian Journal of Geosciences. 2016;9(4). 10.1007/s12517-015-2138-3

[56] Zhu H, Xiubin LI. Discussion on the Index Method of Regional Land Use Change. Acta Geographica Sinica. 2003;58(5):643–50.

[57] Al-sharif AAA, Pradhan B. Monitoring and predicting land use change in Tripoli Metropolitan City using an integrated Markov chain and cellular automata models in GIS. Arabian Journal of Geosciences. 2014;7(10):4291–301. 10.1007/s12517-013-1119-7

[58] Yousheng W, Xinxiao Y, Kangning H, Qingyun L, Yousong Z, Siming S. Dynamic simulation of land use change in Jihe watershed based on CA-Markov model. Transactions of the Chinese Society of Agricultural Engineering. 2011;2011(12). 10.3969/j.issn.1002-6819.2011.12.062

[59] Ma C, Zhang GY, Zhang XC, Zhao YJ, Li HY. Application of Markov model in wetland change dynamics in Tianjin Coastal Area, China. Procedia Environ Sci. 2012;13:252–62. 10.1016/j.proenv.2012.01.024.

[60] Fang-jun LIAO Z D-s. Forestry Landscape Patterns Changes and Dynamic Simulation of Nanling National Nature Reserve, Guangdong. SCIENTIA GEOGRAPHICA SINICA. 2014;34(9):1099–107. 10.13249/j.cnki.sgs.2014.09.1099

[61] El-Hallaq MA, Habboub MO. Using GIS for Time Series Analysis of the Dead Sea from Remotely Sensing Data. Open Journal of Civil Engineering. 2014:386–96. 10.4236/ojce.2014.44033

[62] Mishra VN, Rai PK, Mohan K. Prediction of Land Use Changes Based on Land Change Modeler (LCM) Using Remote Sensing: A Case Study of Muzaffarpur (Bihar), India. Journal of the Geographical Institute Jovan Cvijic Sasa. 2014;64(1):111–27.

[63] Mao JX, Yan XP. Corridor Effects of the Urban Transport Artery on Land Use——A Case Study of the Guangzhou Avenue. Geography and Geo-Information Science. 2004;20(5):58–61.

[64] JING Yunqing ZF, ZHANG Yue. Change and prediction of the land use /cover in Ebinur Lake Wetland Nature Reserve based on CA-Markov model. Chinese Journal of Applied Ecology. 2016;27(11):3649–58. 10.13287/j.1001-9332.201611.027 /j.1001-9332.201611.027. 29696864

[65] Zhang Rongtian JH. Evolution and simulation of land use/land scape pattern in Ning -Zhen—Yang hilly area. Science of Surveying and Mapping. 2016;41(3):85–90. 10.16251/j.cnki.1009-2307.2016.03.017

[66] Xiao M, Wu J, Chen Q, Jin M, Hao X, Zhang Y. Dynamic change of land use in Changhua downstream watershed based on CA-Markov model. Transactions of the Chinese Society of Agricultural Engineering. 2012;28(10):231–8.

[67] Liu SH, Shu-Jin HE. A spatial analysis model for measuring the rate of land use change. Journal of Natural Resources. 2002;17(5):533–40.

[68] Feinstein AR, Cicchetti DV. High agreement but low kappa: I. The problems of two paradoxes. J Clin Epidemiol. 1990;43(6):543–9. 10.1016/0895-4356(90)90158-l 2348207

[69] Cicchetti DV, Feinstein AR. High agreement but low kappa: II. Resolving the paradoxes. J Clin Epidemiol. 1990;43(6):551–8. 10.1016/0895-4356(90)90159-m. 2189948

